# Effects of butylphthalide sodium chloride injection combined with edaravone dexborneol on neurological function and serum inflammatory factor levels in sufferers having acute progressive cerebral infarction

**DOI:** 10.3389/fneur.2024.1415977

**Published:** 2024-12-19

**Authors:** Yaqiang Li, Jie Liu, Yuanyuan Zhu, Min Xue, Jiale He

**Affiliations:** ^1^Department of Neurology, People's Hospital of Lixin Country, Bozhou, China; ^2^Department of Neurology, First Affiliated Hospital of Anhui University of Science and Technology (First People’s Hospital of Huainan), Huainan, China

**Keywords:** edaravone dexborneol, butylphthalide sodium chloride injection, acute progressive cerebral infarction, neurological function, inflammatory factors

## Abstract

**Objectives:**

For investigating an influence on butylphthalide sodium chloride injection combined with edaravone dextromethorphan on neurological function, serum free radical levels, and serum inflammatory factor levels in sufferers having acute progressive cerebral infarction (APCI).

**Methods:**

A cohort of 200 patients, afflicted by APCI and admitted to our healthcare institution between December 2018 through July 2023, were incorporated into this longitudinal prospective analysis. Employing a random number table methodology, the patient cohort was bifurcated into a control group (*n* = 100) and an observation group (*n* = 100). The control group is treated with butylphthalide sodium chloride injection and the study group is treated with edaravone dexborneol, based on the control group. The posttreatment curative efficacy on the two groups is recorded, and treatment of both the two groups is compared. Before and after neurological function indexes (NIHSS and BI), inflammatory factor indexes (hs-CRP, IL-8, and TNF-a), serum free radical levels (ROS, NO, and SOD), and contrasted between the two groups of treatment effect during therapy.

**Results:**

Compared with the control (65%), the observation group exhibited a significantly higher effective rate of around 89%. In the observation group, the improvement in NIHSS, and Barthel index scores; inflammatory indexes including hs-CRP, IL-8, and TNF-a; and serum free radical levels including ROS, NO, and SOD in peripheral blood was better than the control group, with significant difference (all P<0.05).

**Conclusion:**

The clinical efficacy of edaravone dexborneol combined with butylphthalide sodium chloride injection in the treatment of acute progressive cerebral infarction is precise, which can effectively reduce the level of inflammatory factors and serum free radicals in patients, improve the neurological function of patients, and reduce the damage of cerebral cells, and the safety is high.

## 1 Introduction

Acute cerebral infarction (ACI) is a common clinical neurological disease, mostly caused by vascular, blood, haemodynamic abnormalities leading to cerebral artery stenosis and blockage, the onset of the disease is acute and rapid, if the treatment is not timely, it will seriously affect the patient’s prognosis for recovery, leading to sequelae such as hemiparesis, affecting the patient’s normal work and life (1, 2). The pathogenesis of cerebral infarction is a complex process involving various mechanisms such as apoptosis, inflammatory response, and free radical damage (3, 4). This disease often results in disability or death, and most patients with acute cerebral infarction (ACI) experience impaired work ability, language dysfunction, and motor dysfunction. Acute progressive cerebral infarction is a common and serious cerebrovascular disease in cerebral infarction, and about 6 h after the onset of the patient will lead to a stepwise aggravation of the condition due to brain tissue damage and neurological deficits, so it has a high rate of death and disability (5). The pathophysiological changes of cerebral infarction are complex, and treating brain tissue and nerve function damage with a single drug can be challenging to achieve satisfactory results. Currently, there is no specific drug for controlling APCI, and drugs that protect brain tissue, antiplatelet aggregation, anti-inflammatory and free radical scavenging effects are mostly used to prevent further deterioration of the disease (6).

After the occurrence of acute cerebral infarction the body releases a large number of inflammatory mediators, activates the inflammatory cytokine network system, stimulates the inflammatory cascade response or inflammatory waterfall effect at the cellular and subcellular levels, which not only increases the damage to the vascular endothelial cells, but also increases the permeability of the capillaries, leading to microthrombosis and local ischemia (7). The body releases a large amount of excitatory amino acids, nitric oxide, oxygen free radicals and other harmful substances secondary to systemic inflammatory response syndrome, which can cause multiple organ dysfunction syndrome in severe cases, further aggravate the condition, increase the risk of death, and is a risk factor for the poor prognosis of patients with acute cerebral infarction (8). Edaravone dexborneol is a newly-launched national Class I innovative drug, which has the unique effects of scavenging free radicals, inhibiting inflammatory factors and protecting brain cells and nerve tissues (9). Butylphthalide Sodium Chloride Injection is a new drug independently researched and developed in China, which has a variety of effects such as antiplatelet aggregation, improvement of blood supply to the brain and enhancement of cerebral function metabolism (10). A randomised controlled multicentre trial has demonstrated that butylphthalide treatment for 90 days significantly improves cerebral autoregulation in patients with ACI (11). An animal study revealed that Edaravone dexborneol provides neuroprotective benefits by suppressing NLRP3 inflammasome-induced microglial pyroptosis in experimental ischemic stroke (12).

At present, there are no clinical reports on the treatment of APCI with edaravone dexborneol combined with butylphthalide sodium chloride injection, so the present study investigates the clinical efficacy and safety of edaravone dexborneol combined with butylphthalide sodium chloride injection in the treatment of APCI, and provides references for the use of the drug in the clinic.

## 2 Materials and methods

### 2.1 Subjects

Patients with APCI admitted to Department of Neurology, First Affiliated Hospital of Anhui University of Science and Technology (First People’s Hospital of Huainan) from December 2018 through July 2023 were methodically assessed. According to the inclusion and exclusion criteria, the cases that fit this study were selected. The following were the inclusion requirements: (1) Age greater than 40 and less than 80; (2) Presenting with stroke symptoms and receiving treatment within 6 to 48 h; (3) progressive deterioration within 72 h of onset; (4) clinically diagnosed with ACI, corroborated by imaging modalities. The following are the exclusion criteria: (1) patients undergoing intravenous thrombolysis or arterial thrombolysis; (2) patients with intracranial hemorrhage; (3) patients having severe organ dysfunction, including those of the heart, liver, and kidneys; (4) patients suffering from a mental disorder or consciousness dysfunction; (5) patients allergic to the drugs; (6) patients with coagulation dysfunction; and (7) comatose or unconscious patients. Ultimately, a cohort of 200 patients was constituted and randomized into an observation group (*n* = 100) and a control group (*n* = 100). All methodologies used adhered to the Declaration of Helsinki, and institutional approval was obtained from the hospital’s ethics board (approval code: 2018-KY-102-023). Each individual’s consent was documented through signed informed consent forms.

### 2.2 The therapeutic methods

The patients were divided into a control group and an observation group according to the treatment method. All of the patients in both groups received conventional treatment for cerebral infarction, including antiplatelet aggregation, lipid regulation, plaque stabilization, microcirculation improvement, and blood pressure control. The control group is given butylphthalide sodium chloride injection (NBP Pharmaceutical Co, Ltd., of CSPC, specification: 100 mL, Chinese medicine approved word: H22025067) intravenous drip therapy, twice a day, 25 mg (100 mL) each time; each instillation time is not less than 50 min, the interval between the two administrations is not less than 6 h, and the course of treatment is 14 d. Basing on the control group, the observation group is treated combined with concentrated solution of edaravone and dexborneol for injection (Nanjing Xiansheng TECO Pharmaceutical Co., Ltd., specification: 5 mL: 10 mg (edaravone): 2.5 mg (dexborneol), Chinese medicine accurate word: H20200007) intravenous infusion treatment, 15 mL each time (containing edaravone 30 mg, dexborneol 7.5 mg), 2 times a day. When in use, add it to 100 mL of normal saline to dilute it and then infuse it intravenously; finish the drip within 30 min and treat it continuously for 14 days. The drugs were delivered with an identifiable label, i.e., the patients knew which treatment they received.

### 2.3 Laboratory tests

Peripheral venous blood was sampled before and after 14 d of treatment in both groups. Five ml of peripheral phlebo blood is collected from two groups, put in an anticoagulant tube for 1.5 h, and centrifuged with a centrifuge (Hettich, Germany, Rotofix 32A) for 10 min at a velocity of 3,000 r/min; separate the supernatant and save it for later use. The serum was isolated and stored in a refrigerator at −80°C to be ready for later testing. Serum concentrations of tumour necrosis factor-*α* (TNF-α) and interleukin-8 (IL-8) were measured by enzyme-linked immunosorbent assay (ELISA) in both groups of patients before and after treatment. The ELISA kits for TNF-α and IL-8 were purchased from Shanghai Enzyme-linked Biotechnology Company (China). Serum superoxide dismutase (SOD) and reactive oxygen species (ROS) levels were measured using a colourimetric assay, and the kit was purchased from Wuhan Saipei Biotechnology Company. These formulas allowed us to quantify and analyze different aspects of the data. By using these calculations, we were able to assess various factors and draw conclusions about the data set. It is pertinent to note that the investigators responsible for the biomarker concentration assessments operated in a central laboratory and were blinded to the clinical outcomes of the patients under study.

### 2.4 Observation indicators

The neurological function of the two groups before and after treatment was evaluated by the National Institutes of Health Stroke Scale (NIHISS), which consisted of 15 scores, including level of consciousness, command coordination, eye movement, visual field defects, and language expression, with a total score of 42 points (13). The degree of neurological deficit was positively correlated with the score. The Barthel index (BI) scale was used to assess the two groups’ daily living ability, with a range of total scores from 0 to 100 (14). All patients were evaluated for patient NIHISS scores and BI scores before treatment and after 14 days of treatment. The higher the score, the higher the patients’ daily living ability. Although not requiring a trained nurse, but requiring someone to attend several times throughout the day and night. Record and compare the emergence of the untoward effect during treatment, containing vomiting, fever, disturbance of consciousness, convulsions, and mental symptoms.

### 2.5 Statistical analysis

We employed SPSS 26.0 (Inc., Chicago, IL, United States) for our statistical evaluations. Data with a normal distribution was represented as mean ± SD, and the significance of the differences between groups was gauged through the t-test. For non-normally distributed datasets, we used the Mann–Whitney U test, indicating data as median along with its IQR. Categorical data differences, shown as frequency (percentage), were analyzed using chi-square tests. A *p*-value below 0.05 was deemed statistically significant.

## 3 Results

### 3.1 Clinical and demographic characteristics of patients in observation group and control group

The study included a sample of 200 participants, with 100 individuals in the control group (55 of whom were women) and 100 individuals in the observation group (49 of whom were women). The average age of the control group was 68.31 ± 5.32 years, while the average age of the observation group was 66.44 ± 5.03 years. An analysis of the data showed that there was no significant difference in age between the two groups (*p* > 0.05). Likewise, other basic characteristics did not show any statistically significant differences between the control and observation groups, as presented in Table 1.

**Table 1:** Clinical and demographic characteristics of patients in observation group and control group.

Variables	Observation group (*N* = 100)	Control group (*N* = 100)	*p* value
Demographic characteristics			
Gender, female, *n*(%)	55 (55.00)	49 (49.00)	0.396
Age, years, mean ± SD	66.44 ± 5.03	68.31 ± 5.32	0.523
Education years, median (IQR)	6 (3–8)	6 (3–9)	0.467
Married, *n*(%)	94 (94.00)	96 (96.00)	0.516
Vascular risk factors (%)			
Hypertension	75 (75.00)	69 (69.00)	0.345
Diabetes mellitus	31 (31.00)	38 (38.00)	0.298
Coronary heart disease	13 (13.00)	16 (16.00)	0.547
Atrial fibrillation	15 (15.00)	16 (16.00)	0.845
current smoking	23 (23.00)	19 (19.00)	0.487
Alcohol consumption	24 (24.00)	26 (26.00)	0.744
SBP (mmHg)	152.21 ± 8.45	150.98 ± 9.21	0.843
DBP (mmHg)	87.77 ± 8.69	88.21 ± 6.25	0.763
Laboratory parameters (IQR)			
TG, mmol/L, median (IQR)	1.29 (0.87–1.81)	1.28 (0.84–2.31)	0.342
TC, mmol/L, median (IQR)	4.32 (3.54–5.53)	4.39 (3.45–5.51)	0.437
HDL-C, mmol/L, median (IQR)	1.17 (0.79–1.31)	1.08 (0.79–1.32)	0.523
LDL-C, mmol/L, median (IQR)	2.46 (1.98–3.17)	2.23 (1.86–3.32)	0.432
ApoA, g/L, median (IQR)	1.15 (1.09–1.52)	1.35 (1.11–1.51)	0.823
ApoB, g/L, median (IQR)	0.73 (0.71–1.32)	0.84 (0.69–1.10)	0.641
Glucose, mmol/L, median (IQR)	5.25 (4.65–6.73)	5.30 (4.64–6.87)	0.798
TOAST subtype (*n*.%)			0.309
Large artery atherosclerosis	47 (59.17)	53 (44.17)	-
Cardioembolism	15 (10)	19 (12.5)	-
Small vessel occlusion	38 (40.83)	28 (43.33)	-

### 3.2 Comparative analysis of the serum levels of hs-CRP, IL-8, and TNF-*α* between the groups before and after treatment

**Figure 1:**
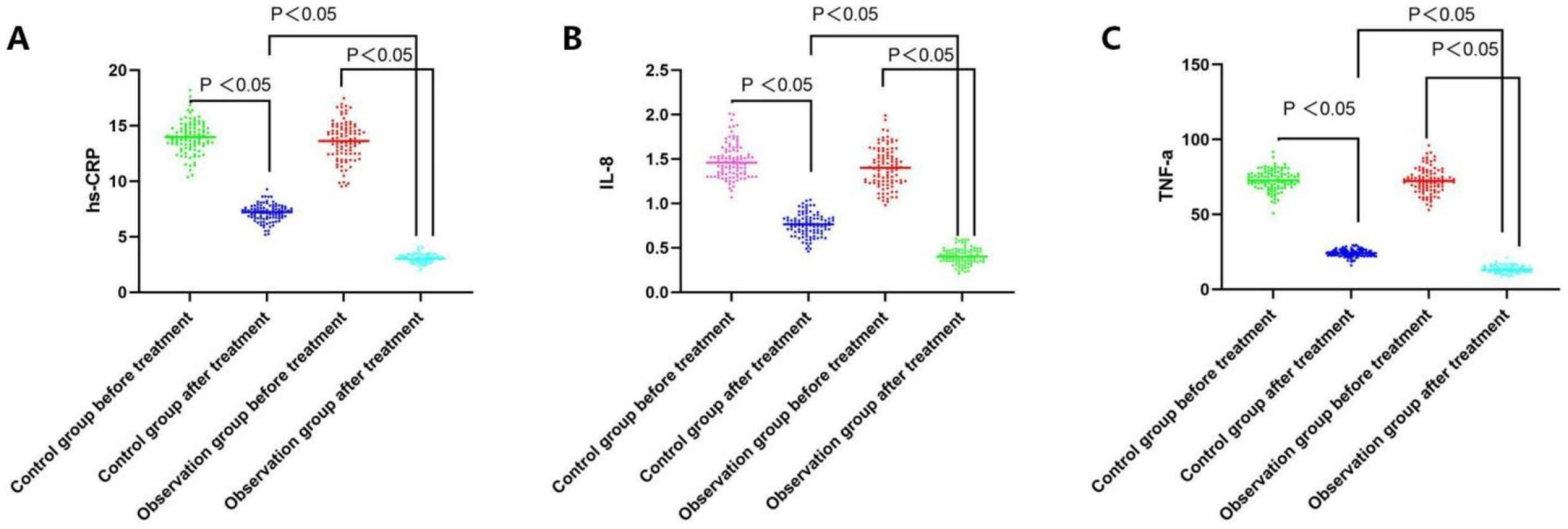
Comparative analysis of the hs-CRP, IL-8, and TNF-a level before and after treatment between the groups. **(A)** Serum hs-CRP levels of both groups before and after the treatment; **(B)** Serum IL-8 levels of both groups before and after the treatment; **(C)** Serum TNF-a levels of both groups before and after the treatment.

Prior to treatment, the levels of hs-CRP, IL-8, and TNF-*α* did not exhibit a significant difference between the groups (all *p* > 0.05). Following treatment, serum hs-CRP, IL-8, and TNF-α levels decreased in both groups, and the improvement degree of serum hs-CRP, IL-8, and TNF-α was greater in the observation group than in the control group (all *p* < 0.05, Table 2; Figure 1).

**Table 2:** Comparative evaluation of serum levels of hs-CRP, IL-8, and TNF-α between the groups before and after treatment.

Variables	Control group (*n* = 100)		Observation group (*n* = 100)	
	Before treatment	After treatment	Before treatment	After treatment
hs-CRP (mg/L)	13.67 ± 1.45	7.15 ± 0.87	13.48 ± 1.65	3.09 ± 0.37
IL-8 (ng/L)	1.46 ± 0.19	0.76 ± 0.11	1.44 ± 0.21	0.40 ± 0.08
TNF-α (ng/L)	72.41 ± 6.73	23.72 ± 2.60	73.02 ± 8.79	13.26 ± 2.41

hs-CRP: high sensitivity C-reactive protein; IL-8: Interleukin-8; TNF-α: Tumor necrosis factor-α; Compared with before treatment in the same group, *P < 0.05; Compared with control group in the same time, #*p* < 0.05.

### 3.3 Comparative analysis of the ROS, NO, and SOD levels in peripheral blood before and after treatment in the two groups

**Figure 2:**
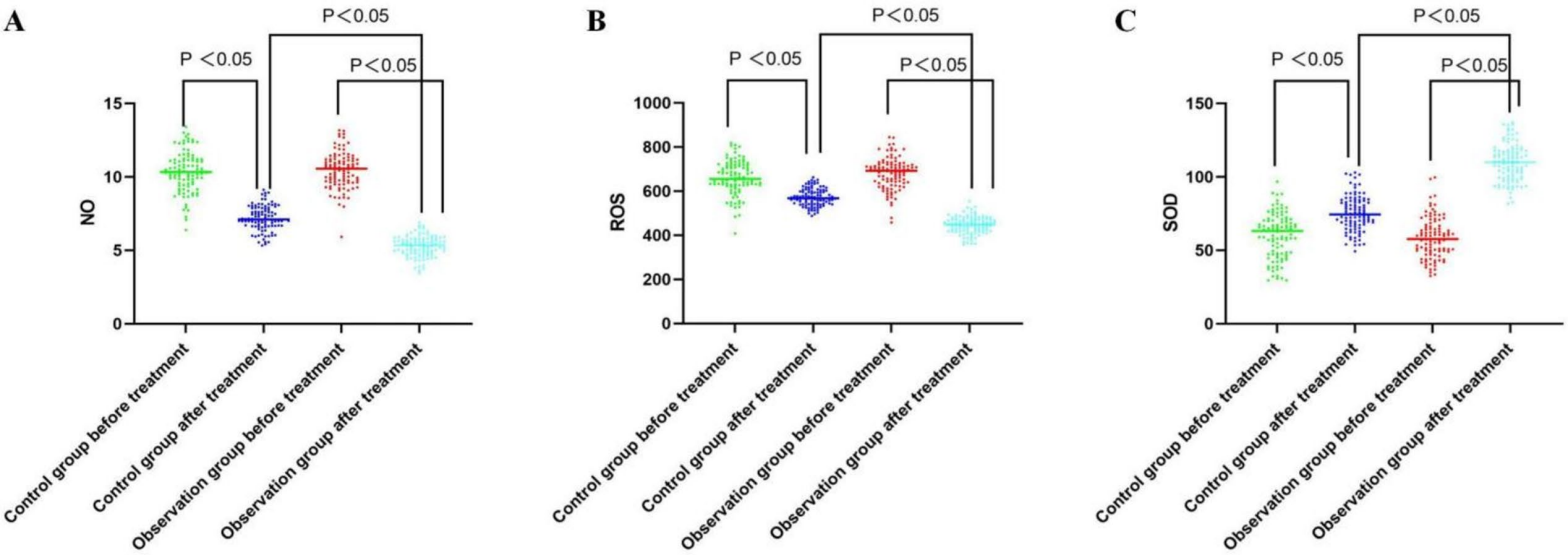
Comparative analysis of the NO, ROS, and SOD level before and after treatment between the groups. **(A)** Serum NO levels of both groups before and after the treatment; **(B)** Serum ROS levels of both groups before and after the treatment; **(C)** Serum SOD levels of both groups before and after the treatment.

Peripheral blood levels of ROS, NO, and SOD were not significantly different between the groups prior to the treatment (all *p* > 0.05). Peripheral blood levels of SOD increased in both groups following treatment, while ROS and NO levels dropped in both groups. While in the observation group, greater improvement of ROS, NO, and SOD levels in peripheral blood was observed as compared to the control group (all *p* < 0.05, Table 3; Figure 2).

**Table 3:** Comparative analysis of the ROS, NO, and SOD levels in peripheral blood before and after treatment in the two groups.

Variables	Control group (*n* = 100)		Observation group (*n* = 100)	
	Before treatment	After treatment	Before treatment	After treatment
ROS, μmol/L	661.30 ± 75.87	573.75 ± 39.38	675.01 ± 74.43	448.93 ± 38.09
NO, μmol/L	10.33 ± 1.25	7.08 ± 0.97	10.48 ± 1.31	5.23 ± 0.81
SOD, μmol/L	59.64 ± 15.91	75.28 ± 12.35	58.37 ± 14.62	110.64 ± 13.01

ROS: Reactive oxygen species; NO: Nitric oxide; SOD: Superoxide dismutase orgotein; Compared with before treatment in the same group, *p < 0.05; Compared with control group in the same time, #*p* < 0.05.

### 3.4 Comparison of NIHISS and Barthel index scores between the two groups

In the control group, the scores of patients’ NIHSS before and after treatment were [9 (8-12)] and [6 (4-8)], respectively. Similarly, the BI scores were [45 (40–60)] and [45 (35–75)], respectively. In the cohort under observation, the NIHSS scores before and after treatment were [9 (8-12)] and [5 (4-7)], correspondingly; likewise, the BI scores exhibited similar patterns, reflecting [45 (35–65)] and [55 (45–85)]. The information presented in Table 4 revealed no statistically significant variation in NIHSS and BI scores between the groups upon initial admission (*p* > 0.05). After the intervention, the experimental group exhibited reduced NIHSS scores and improved BI scores compared to the control group, with a significant statistical difference (*p* < 0.05).

**Table 4:** Comparison of NIHSS scores and barthel index score between the two groups.

Group	NIHSS scores		Barthel index score	
	Before treatment	After treatment	Before treatment	After treatment
Observation group	9 (8–12)	5 (4–7)	45 (35–65)	55 (45–85)
Control group	9 (8–12)	6 (4–8)	45 (40–60)	45 (35–75)
*P* value	0.324	0.019	0.148	0.042

### 3.5 Comparison of the treatment effect of the two groups

In the control group, 16 patients recovered basically, 19 patients were significantly effective, 30 patients were effective, 33 patients were ineffective and 2 patients deteriorated. Conversely, the observation cohort reported 24 cases of basically recovered, 29 cases of significant effect, 36 patients were effective, 10 patients were ineffective and 1 patients deteriorated. These data, depicted in Table 5, substantiate that the control cohort had a lower overall effectiveness rate, achieving statistical significance (*p* < 0.05).

**Table 5:** Comparison of the treatment effect of the two groups (*n*, %).

Group	Control (*n* = 100)	Observation (*n* = 100)	χ2 value	*P*-value
Basically recovered	16 (16.00)	24 (24.00)	-	-
Significant effect	19 (19.00)	29 (29.00)	-	-
Effective	30 (39.00)	36 (36.00)	-	-
Invalid	33 (33.00)	10 (10.00)	-	-
Deterioration	2 (2.00)	1 (1.00)	-	-
Total effective rate	65 (65.00)	89 (89.00)	16.262	0.000

## 4 Discussion

It is well known that acute progressive cerebral infarction is a common acute ischaemic cardiovascular and cerebrovascular disease with rapidly worsening symptoms and complex pathogenesis. APCI is a special type of cerebral infarction in which the patient’s condition continues to progress after conventional thrombolytic and antiplatelet treatments, with continued apoptosis of brain cells, severe impairment of neurological function, and a high rate of morbidity and mortality. Currently, there is no effective treatment for APCI, so how to delay the further deterioration of APCI and inhibit apoptosis to improve the clinical prognosis is the current research hotspot for this condition. Butanephthalein Sodium Chloride Injection is a new drug independently developed by our country, which has the effect of improving the blood supply of brain tissues and microcirculation, shrinking brain foci, reducing the oxidative damage of brain cells and improving the neurological function. Its efficacy and safety have been evaluated in several clinical trials conducted in China (15, 16). Recently in China, a noteworthy neuroprotective compound known as edaravone dexborneol has been synthesized. This compound is composed of (+)-borneol and edaravone in a 1:4 ratio (17).

Studies in Phase II and III have revealed that Chinese individuals who received edaravone dexborneol treatment within 48 h of experiencing acute ischemic stroke exhibited superior functional outcomes in comparison to those treated solely with edaravone. Additionally, edaravone dexborneol has demonstrated a favorable safety profile and tolerance across various dosages when compared with edaravone alone (17, 18). Since July 2020, the National Medical Products Administration (NMPA) of China has granted clearance for clinical application of edaravone dexborneol in AIS patients. This treatment has shown promising results. It is believed that the neuroprotective effects of edaravone dexborneol against AIS are mediated through a versatile cytoprotective pathway, encompassing various aspects such as oxidative stress, excitotoxicity, inflammation, and apoptosis. The combination of their respective advantages can inhibit the inflammatory reaction caused by cerebral ischemia/reperfusion, prevent the interaction between oxygen free radicals and inflammatory cytokines, enhance the protection of brain cells, improve the permeability of the blood–brain barrier, and reduce the damage of neurological function.

The findings of this study revealed that the clinical efficacy of edaravone dextroamphetamine combined with butylphthalide sodium chloride injection in the treatment of patients with APCI was significantly better than that of butylphthalide sodium chloride injection alone, which was similar to the results of the study by Li et al. (19). In addition, edaravone dextroamphetamine could better inhibit the expression of inflammation-related factors and play a synergistic effect with butalphalein sodium chloride injection. The neurovascular unit is composed of neurons, microvessels and microglia, which control the exchange of substances between the blood and brain and stabilise the internal environment of the brain. However, when studying cardiovascular and cerebrovascular diseases in the past, neuronal and cellular populations were the focus of research, while the holistic nature of the brain’s neurological function was often overlooked, and in the event of a cerebral infarction, there is severe damage to the neurovascular unit. In addition, excess free radicals in the body can also cause secondary brain damage, resulting in further damage to brain tissue.

The outcome of this investigation revealed that the degree of reduction of NIHSS score and the degree of increase of BI score of the observation group were better than that of the control group; the serum ROS and NO levels of the observation group were lower than those before treatment, and the SOD level was higher than that before treatment, and the magnitude of change was higher than that of the control group, suggesting that the combination of drugs can significantly improve the nerve function of patients and reduce the level of serum free radicals. Dexamethasone can rapidly capture excess free radicals and increase the activity of SOD in ischaemic neurons, thus inhibiting free radicals. In addition to inhibiting oxidative stress, edaravone dexamethasone can inhibit neuronal apoptosis under oxidative stress caused by various etiological factors, and has a protective effect on the entire neurovascular unit (20). Butylphthalide sodium chloride injection can protect the integrity of the mitochondrial structure and maintain the stability of the mitochondrial membrane, thus maintaining energy metabolism and reducing neuronal cell death, which is consistent with the findings of Zhang et al. (21).

After ACI, the lesion will release a large number of inflammatory factors. hs-CRP, IL-8 and TNF-*α* are typical inflammatory factors in the development of infarction patients, which can induce neuronal cell apoptosis, destroy the coagulation mechanism, and exacerbate the patient’s condition, so that the detection of inflammatory factors can accurately reflect the severity of the patients with acute progressive cerebral infarction. Wu et al. further demonstrated that the combination of edaravone and dexamethasone could significantly alleviate ischemic injury and produce neuroprotection by inhibiting ischemia/reperfusion-induced TNF-*α* expression and exerting anti-inflammatory properties (22). In addition, butylphthalide sodium chloride injection significantly attenuated microglial cell activation and elevated levels of proinflammatory cytokines after stroke, and demonstrated improved neurologic function (23). The findings of this study demonstrated that the levels of hs-CRP, IL-8, and TNF-α decreased in both groups, and the decrease in the observation group was higher than that in the control group, suggesting that the combination of the two drugs can significantly inhibit the expression of inflammatory factors. Dexamphetamine, an inflammatory factor inhibitor, can inhibit the expression of a variety of inflammatory factors, and its anti-inflammatory effect is more significant when used in combination with butylphthalide sodium chloride injection, which is consistent with the results of the study by Shen et al. (24).

## 5 Conclusion

In conclusion, edaravone dextromethorphan combined with butylphthalide sodium chloride injection can effectively improve the neurological function of patients with acute progressive cerebral infarction, reduce the level of serum free radicals and inflammatory factors, attenuate brain damage, and play a protective role in neurons, with a high degree of safety.

## Data Availability

The original contributions presented in the study are included in the article/supplementary material, further inquiries can be directed to the corresponding author.
